# Noise Fingerprints as a Quantitative Order Parameter for Polarization‐ and Defect‐Mediated Switching in Hafnia Ferroelectrics

**DOI:** 10.1002/advs.202519391

**Published:** 2026-02-08

**Authors:** Ryun‐Han Koo, Jiseong Im, Joon Hwang, Sung‐Ho Park, Jonghyun Ko, Kangwook Choi, Sangwoo Ryu, Gyuweon Jung, Jong‐Ho Lee

**Affiliations:** ^1^ Department of Electrical and Computer Engineering and Inter‐University Semiconductor Research Center (ISRC) Seoul National University Seoul South Korea; ^2^ SK Hynix Inc Icheon Republic of Korea

**Keywords:** defect‐mediated switching, hafnia ferroelectrics, low‐frequency noise, noise fingerprints, noise spectroscopy, polarization‐mediated switching

## Abstract

Noise, often regarded as an unwanted background in electronic devices, can instead serve as a sensitive probe of switching dynamics. In hafnia ferroelectrics, the coexistence of polarization‐mediated (P‐RS) and defect‐mediated (D‐RS) resistance switching has been widely debated, yet prior evidence has remained qualitative. Here, we demonstrate that low‐frequency noise (LFN) can be transformed into a quantitative order parameter that disentangles the two mechanisms across program bias (*V*_PGM_) and processing conditions. By varying the O_3_ dose time during HfZrO atomic layer deposition (ALD), the normalized power spectral density (*S*_I_/*I* ^2^) consistently exhibits a rise–peak–fall profile. We introduce a deconvolution framework, grounded in monotonic baselines and alternating projections, to extract physically consistent polarization‐ and defect‐mediated current components. The resulting process‐bias maps show that reduced O_3_ dose shifts the P‐RS/D‐RS crossover to lower *V*_PGM_ and sharpens the transition, directly linking oxygen stoichiometry to the competition between switching pathways. This quantitative approach resolves the long‐standing controversy over P‐RS and D‐RS coexistence and provides practical guidance for the design of hafnia‐based ferroelectric devices.

## Graphical abstract

Low‐frequency noise fingerprints in hafnia ferroelectrics provide a quantitative handle to resolve the long‐standing debate between polarization‐mediated and defect‐mediated switching. By tuning oxygen vacancy density via ALD O3 dose time and applying a physically constrained deconvolution, we extract bias‐resolved current fractions for both mechanisms and map their crossover. The method offers practical, process‐guided design rules for hafnia‐based devices.

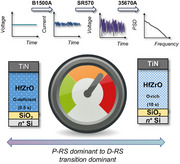

## 1 Introduction

Noise has long been considered a nuisance in electronic devices, but it is increasingly recognized as a powerful probe of hidden dynamics in materials and devices. In particular, low‐frequency noise (LFN), expressed as the normalized power spectral density (*S*_I_/*I* ^2^), captures fluctuations from carrier trapping, defect activity, and ionic motion that remain invisible in conventional current–voltage measurements [1, 2, 3, 4, 5, 6]. Because of this sensitivity, noise spectroscopy has been widely applied to study reliability in complementary metal‐oxide‐semiconductor (CMOS) transistors, charge‐trap memories, resistive RAM (RRAM), ferroelectrics, and emerging low‐dimensional semiconductors [7, 8, 9, 10, 11, 12, 13]. Beyond reliability diagnostics, recent ferroelectric‐memory studies have also leveraged device uncertainty/noise as a computational resource for Bayesian or uncertainty‐aware inference [14, 15]. In contrast, the present work uses low‐frequency noise as a mechanism‐resolving probe and introduces a quantitative deconvolution framework to separate concurrent polarization‐ and defect‐mediated contributions under controlled vacancy tuning. Hafnium‐oxide ferroelectrics (HfO_2_ and its alloys such as HfZrO) have gained significant attention as CMOS‐compatible thin films that maintain robust ferroelectricity at nanometer scales. They combine fast switching, low operating voltage, and strong endurance, making them promising candidates for non‐volatile memories, logic circuits, and neuromorphic systems [16, 17, 18, 19].

Despite this promise, the microscopic mechanisms governing switching in HfO_2_‐based ferroelectrics remain unresolved and highly debated. Two principal contributions are discussed in the literature: (i) polarization‐mediated resistance switching (P‐RS), in which ferroelectric polarization reversal modulates the barrier profile and conduction; and (ii) defect‐mediated resistance switching (D‐RS), in which oxygen vacancy redistribution modifies the effective barrier height or width [20, 21, 22, 23, 24]. Both P‐RS and D‐RS can generate hysteretic signatures that typically appear as anticlockwise loops in current–voltage (*I*–*V*) sweeps. They may also coexist in the same device, which makes them difficult to distinguish. Because P‐RS and D‐RS can both yield similar *I*–*V* hysteresis and tunneling electroresistance (TER) ratio trends, especially when polarization switching and vacancy‐assisted conduction coexist under high‐field programming, mechanism assignment from electrical sweeps alone often remains ambiguous and largely qualitative. In particular, the dual role of oxygen vacancies is particularly controversial: they are believed to stabilize the polar orthorhombic phase [25, 26], while simultaneously providing the mobile species responsible for ionic conduction [27, 28]. This duality has become a central point of controversy in the field, as researchers still lack consensus on which switching events are genuinely ferroelectric and which are dominated by defect motion.

Earlier studies from our group suggested that noise could help resolve this ambiguity. However, our prior studies [30, 31, 32] mainly provided qualitative or semi‐quantitative identification (e.g., TER/polarization trends and noise fingerprints) and did not extract a bias‐resolved partitioning of the total current into concurrent P‐RS and D‐RS contributions. We observed that the *S*_I_/*I* ^2^ behaves oppositely in the two regimes: it increases with bias in polarization‐mediated switching and decreases in defect‐mediated switching [29, 30, 31, 32]. These findings provided only indirect, qualitative evidence that noise reflects the underlying mechanism. However, such observations could not reveal how much of the switching is P‐RS driven and how much is D‐RS driven, nor could they determine the precise crossover bias at which the dominance shifts from P‐RS to D‐RS. In other words, while noise trends hinted at a diagnostic role, there was no framework to translate them into quantitative fractions or to locate the transition point reproducibly. Moreover, prior analyses were limited to secondary process variables, such as annealing temperature or interface traps, rather than directly tuning the oxygen vacancy concentration. As a result, the impact of controlled oxygen stoichiometry on the balance between P‐RS and D‐RS has remained unexplored. This critical gap has motivated the present work.

In this work, we address this gap by directly varying the oxygen‐vacancy density through controlled O_3_ dose time during HfZrO atomic layer deposition (ALD), and by establishing a quantitative deconvolution framework for noise in HfO_2_‐based ferroelectric films. Rather than relying on indirect electrical hysteresis, we directly fit the bias‐dependent noise spectra to recover the polarization fraction *x*(*V*_PGM_) = *I*_P_/*I* (polarization‐mediated current fraction) and its complement (*I*_D_/*I*, defect‐mediated current fraction), together with the absolute current components *I*_P_(*V*_PGM_) and *I*_D_(*V*_PGM_) and their associated noise powers, under physically grounded constraints such as monotonic baselines, alternating projections, and current conservation. This provides, for the first time to our knowledge, a quantitative determination of how much of the switching is polarization‐driven versus defect‐driven across the full program‐bias window.

We further show that the crossover point (*x* = 0.5, where polarization and defect contributions are equal) shifts systematically to lower *V*_PGM_ with reduced O_3_ dose. This establishes a direct, quantitative criterion linking oxygen stoichiometry to the onset of the shift from a P‐RS‐dominant regime to a D‐RS‐dominant regime, thereby resolving the long‐standing controversy over their coexistence and providing practical guidelines for engineering next‐generation ferroelectric memories and logic devices.

## 2 Results and Discussion

### 2.1 Material Characterization and Oxygen Stoichiometry Control

**FIGURE 1:**
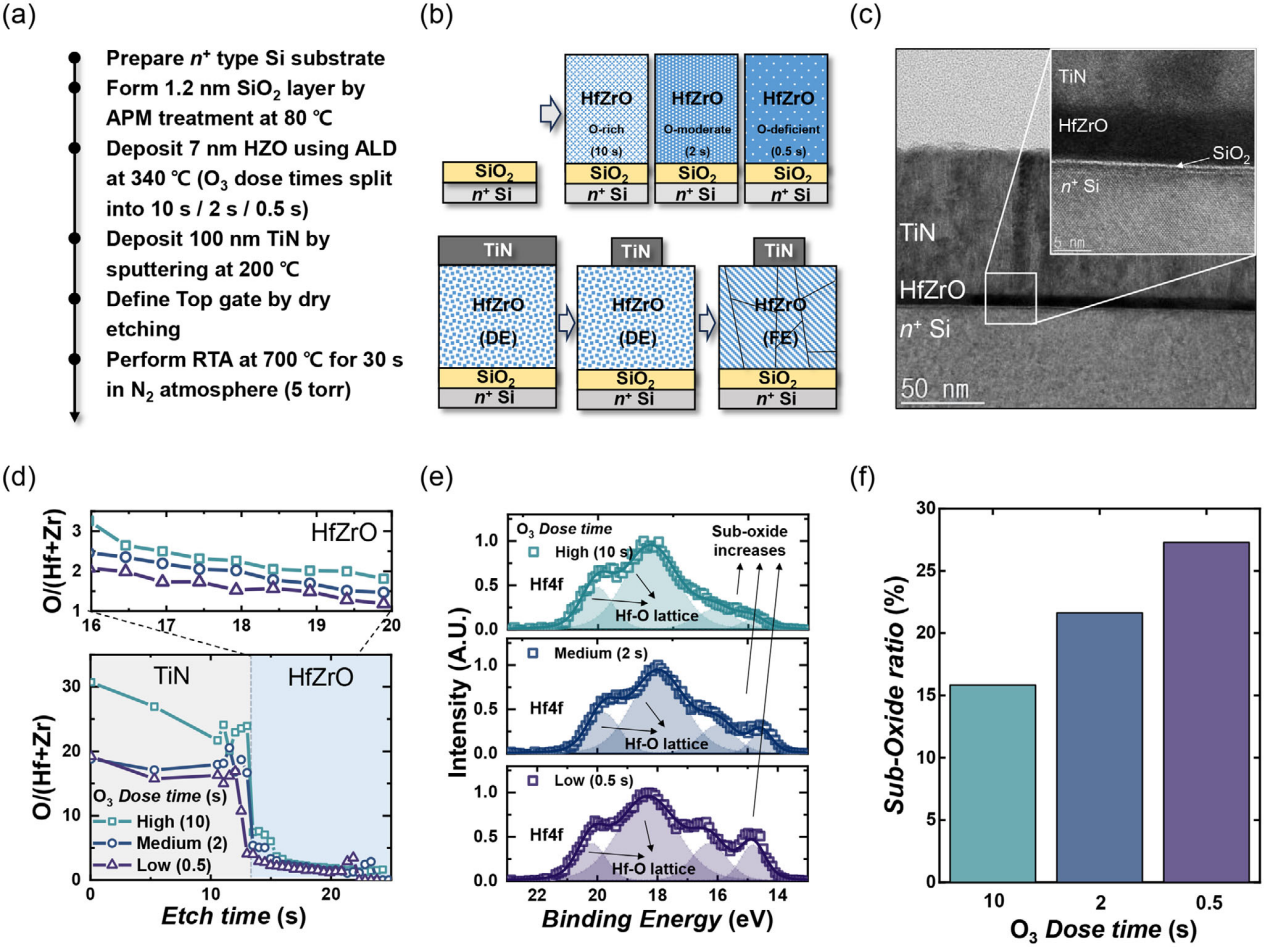
Fabrication of TiN/HfZrO/SiO_2_/*n* ^+^ Si ferroelectric tunnel junctions under different O_3_ dose conditions. (a) Process flow of atomic layer deposition (ALD). (b) Schematic diagram of the device fabrication sequence, showing O‐rich (10 s), O‐moderate (2 s), and O‐deficient (0.5 s) ozone exposure conditions. (c) Cross‐sectional TEM image of the TiN/HfZrO/SiO_2_/*n* ^+^ Si stack (scale bar = 50 nm). Inset: magnified interface region highlighting TiN, HfZrO, and SiO_2_ layers (scale bar = 5 nm). (d) Depth‐profiled O/(Hf+Zr) ratio versus etch time for high (10 s), medium (2 s), and low (0.5 s) O_3_ dose; the upper panel enlarges the HfZrO region. (e) XPS spectra for each O_3_ dose time, deconvolved into four Gaussian components, all shown as shaded regions; the relative intensity of the sub‐oxide component grows as O_3_ dose decreases. (f) Sub‐oxide ratio versus O_3_ dose time, extracted from the fitted Gaussian peak areas.

In this study, we investigate the relative roles of P‐RS and D‐RS in hafnia‐based ferroelectrics. The balance between these two mechanisms is dictated by the density of oxygen vacancies (V_o_), which play a dual role in hafnium oxide: they are required to stabilize the polar orthorhombic phase that enables ferroelectricity, but under bias, they can also migrate and induce defect‐mediated resistive switching. Controlling the V_o_ concentration is therefore a direct route to steer the switching behavior. In this context, we used the O_3_ dose time during the atomic layer deposition (ALD) of HfZrO as a simple process knob to tune the oxygen stoichiometry at the point of film growth, providing a reproducible comparative basis to disentangle P‐RS from D‐RS. Figure 1a summarizes the fabrication and stack structure used in this work. Detailed fabrication procedures are provided in the Section 4 [33]. Note that the Hf and Zr precursor dose times were fixed, and only the O_3_ dose time was varied to control stoichiometry (Figure 1a). Such metal–ferroelectric–insulator–semiconductor (MFIS) stacks are among the most widely adopted structures in both ferroelectric tunnel junctions (FTJs) and ferroelectric field‐effect transistors (FeFETs) [34, 35, 36]. The schematic in Figure 1b contrasts three process conditions: O‐rich (10 s), O‐moderate (2 s), and O‐deficient (0.5 s). A cross‐sectional TEM image of the TiN/HfZrO/SiO_2_/*n* ^+^ Si stack is shown in Figure 1c, and the inset shows a magnified view. Surface morphology (AFM) and grain size statistics of the HfZrO film are provided in Figure S1 (*R*_q_ ≈ 1.87 nm, *R*_a_ ≈ 1.46 nm). Across the three O_3_ dosing conditions, the fabricated devices thus provide a reproducible basis for analyzing the noise fingerprints of the switching mechanisms.

To verify how the O_3_ dose time during ALD affects the stoichiometry of the HfZrO stack, we employed depth‐resolved X‐ray photoelectron spectroscopy (XPS). Details of the XPS sample preparation and data analysis are provided in the Section 4 [37]. As a result, Figure 1d shows the depth‐profiled O/(Hf+Zr) ratio as a function of etch time for devices grown with O_3_ exposures of 10, 2, and 0.5 s. The sharp drop at shallow depth corresponds to the TiN electrode, while the plateau at greater depth reflects the HfZrO layer. In the enlarged upper panel of the HfZrO region, the O/(Hf+Zr) ratio is seen to systematically increase with longer O_3_ exposure, indicating that extended dosing drives the composition closer to stoichiometric with fewer deficiencies. This confirms that reducing the O_3_ dose effectively increases the V_o_ concentration in the films. To further analyze the spectral features, the binding energy scale was calibrated to the C─H component of the C 1s peak at 284.8 eV. A Shirley background was subtracted over the 23–13 eV window, and the intensities were normalized to the maximum of the Hf 4f7/2 peak. Based on this procedure, Figure 1e presents representative spectra of the HfZrO films, deconvolved into two Gaussian doublets. The lattice‐related Hf 4f doublet is observed within 17.5–18.5 eV (4f7/2) and 19.5–20.5 eV (4f5/2), whereas weaker components appear in the 14.5–15.0 eV and 15.5–16.5 eV windows, consistent with sub‐oxide states [38, 39]. The shaded peaks highlight the fitted contributions. The relative weight of the sub‐oxide doublet increases as the O_3_ dose time decreases, showing that shorter exposures produce more oxygen‐deficient bonding configurations. Figure 1f quantifies this effect by plotting the extracted sub‐oxide ratio as a function of O_3_ dose time. The monotonic increase with decreasing exposure confirms that V_o_ defects become more dominant under low‐dose conditions, directly linking the deposition process to the defect‐mediated component of resistance switching.

### 2.2 Electrical Characteristics of P‐RS and D‐RS

**FIGURE 2:**
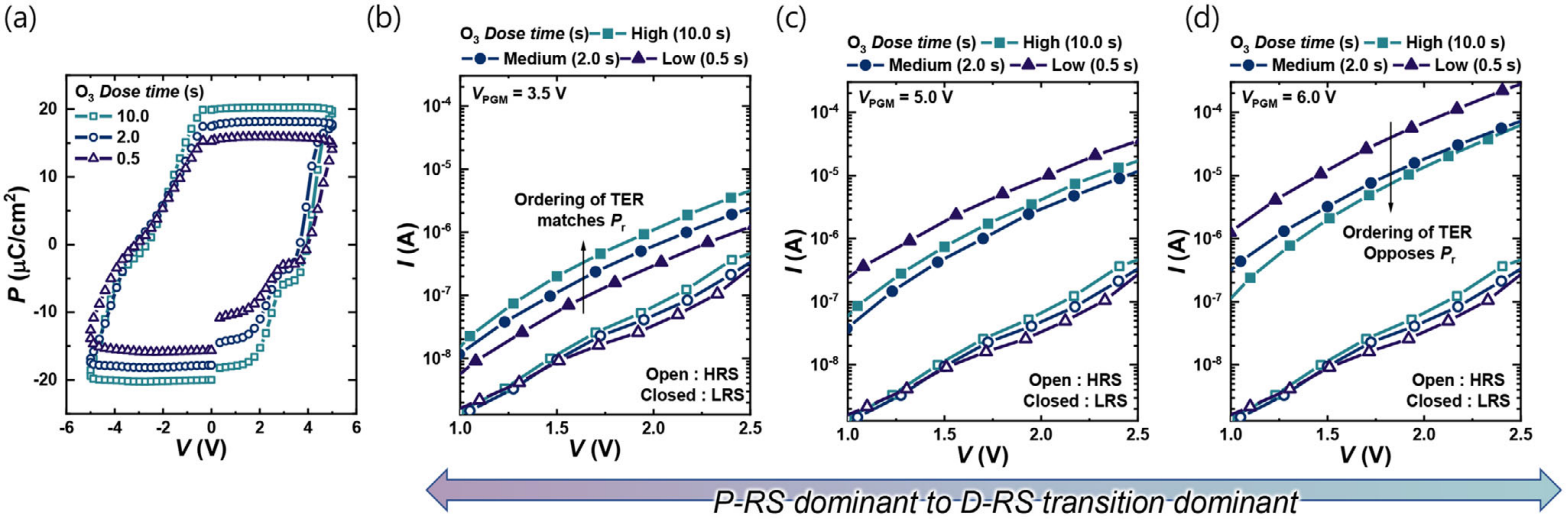
Electrical characteristics of TiN/HfZrO/SiO_2_/*n* ^+^ Si devices under different O_3_ dose conditions. (a) *P*–*V* hysteresis loops for devices fabricated with different O_3_ dose times. (b) *I*–*V* characteristics at *V*_PGM_ = 3.5 V; open symbols denote HRS and closed symbols denote LRS. At this bias, the ordering of TER ratios follows the same trend as *P*_r_. (c) *I*–*V* characteristics at *V*_PGM_ = 5.0 V; open symbols denote HRS and closed symbols denote LRS. (d) *I*–*V* characteristics at *V*_PGM_ = 6.0 V; open symbols denote HRS and closed symbols denote LRS. At this higher bias, the ordering of TER ratios is reversed compared to the *P*_r_ sequence.

Figure 2 presents the electrical characteristics of TiN/HfZrO/SiO_2_/*n* ^+^ Si devices fabricated under different O_3_ dose conditions. Figure S2a shows the PUND measurement scheme using voltage pulses (ramp: 10 µs, top: 1 µs, ±5 V) together with the corresponding transient current responses (Figure S2b–d), which separate polarization‐switching currents from capacitive and leakage components. The resulting polarization–voltage (*P*–*V*) loops in Figure 2a reveal that longer O_3_ exposure yields larger remanent polarization (*P*_r_), indicating enhanced ferroelectricity. This trend is consistent with previous reports that oxygen‐rich conditions suppress excessive V_o_ defects and stabilize the orthorhombic ferroelectric phase of HfO_2_‐based films [40, 41]. However, we emphasize that the *P*_r_–vacancy relationship in hafnia ferroelectrics is process‐window dependent and not universally monotonic; depending on the defect regime, vacancy modulation can either increase or decrease the measured switchable polarization. Therefore, we describe the above *P*_r_ trend as an observation within our specific ALD O_3_‐dose window, rather than a general rule [42, 43, 44, 45]. The dependence of *P*_r_ on O_3_ dose time thus provides indirect confirmation that tuning the ALD O_3_ exposure is an effective way to control V_o_ concentration in the films. Endurance characteristics (*2*P_r_ versus cycle number) for each O_3_ dose are provided in Figure S3. For these measurements, the erase pulse was set to *V*_ERS_ = −5 V with a pulse width of 1 ms. To further examine how O_3_ dose time influences the balance between P‐RS and D‐RS, *I*–*V* characteristics were measured as a function of program voltage (*V*_PGM_) (Figure 2b–d). At a relatively low program voltage of *V*_PGM_ = 3.5 V, the TER ratios follow the same sequence as *P*_r_: devices with stronger ferroelectric polarization also exhibit larger TER (Figure 2b). This indicates that switching in this regime is dominated by polarization‐mediated processes (P‐RS). In ferroelectric tunnel junctions, a larger *P*_r_ enhances the tunneling asymmetry through electrostatic modulation of the barrier, which directly leads to higher TER. At an intermediate program voltage of *V*_PGM_ = 5.0 V, the TER ordering no longer strictly follows either *P*_r_ or the oxygen‐deficiency trend, indicating a mixed regime where P‐RS and D‐RS coexist (Figure 2c). In contrast, at *V*_PGM_ = 6.0 V, the TER ordering is inverted relative to *P*_r_, and oxygen‐deficient devices (short O_3_ dose time) exhibit the largest TER (Figure 2d). This inversion suggests the increasing dominance of D‐RS at higher bias. In vacancy‐rich films, migrating V_o_ defects locally reduce the barrier height and create conductive pathways, which strongly amplify TER. Moreover, the TER values in this regime exceed those at low *V*_PGM_, implying that D‐RS contributions can significantly boost the TER ratio beyond the intrinsic ferroelectric response.

These contrasting behaviors at low and high *V*_PGM_ establish the coexistence of polarization and defect‐mediated mechanisms in HfZrO‐based devices, with their relative weights governed by both process‐induced V_o_ concentration and applied bias. While conventional electrical measurements capture this qualitative shift, they cannot quantitatively disentangle the contributions of P‐RS and D‐RS. To resolve this ambiguity, we turn to LFN spectroscopy, which is highly sensitive to stochastic carrier dynamics and defect motion and thus provides distinctive fingerprints of the active switching mechanism even when the average *I*–*V* curves appear similar.

### 2.3 Low‐Frequency Noise Fingerprints and Transition Metrics

**FIGURE 3:**
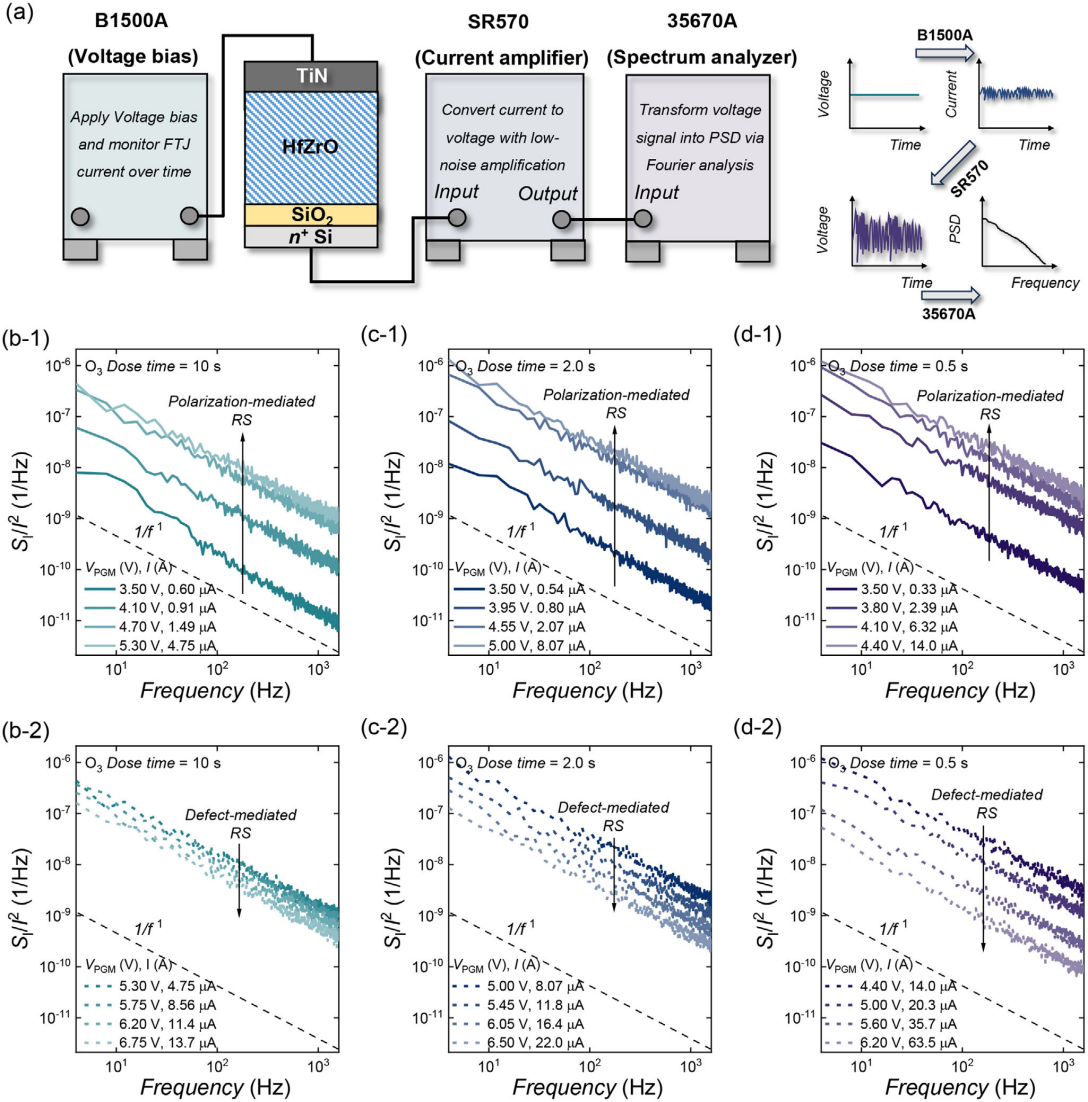
Low‐frequency noise spectra of TiN/HfZrO/SiO_2_/*n* ^+^ Si FTJs under different O_3_ dose times. (a) Schematic of the LFN measurement setup. (b) O_3_ dose time = 10 s. (b‐1) *V*_PGM_ = 3.5 to 5.3 V. (b‐2) *V*_PGM_ = 5.35 to 6.75 V. (c) O_3_ dose time = 2.0 s. (c‐1) *V*_PGM_ = 3.5 to 5.0 V. (b‐2) *V*_PGM_ = 5.0 to 6.5 V. (d) O_3_ dose time = 0.5 s. (d‐1) *V*_PGM_ = 3.5 to 4.4 V. (d‐2) *V*_PGM_ = 4.4 to 6.2 V. In all cases, the polarization‐mediated RS regime exhibits increasing noise with *V*_PGM_, while the defect‐mediated RS regime exhibits decreasing noise with *V*_PGM_.

**FIGURE 4:**
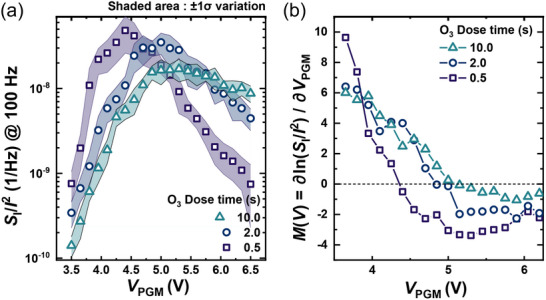
Noise‐fingerprint metrics versus program voltage for different O_3_ dose times. (a) *S*_I_/*I* ^2^ versus *V*_PGM_ for high (10 s), medium (2 s), and low (0.5 s) O_3_ dose; symbols denote the mean traces and the shaded bands indicate ±1σ variation. All cases exhibit a clear maximum defining V*, and decreasing O_3_ shifts V* to lower *V*_PGM_ with a steeper post‐peak roll‐off. (b) Slope metric *M*(*V*) for the same datasets, transitioning from *M* > 0 (polarization‐mediated regime) to *M* < 0 (defect‐mediated regime). The zero‐crossing of *M*(*V*) moves to lower *V*_PGM_ as O_3_ dose time decreases.

**FIGURE 5:**
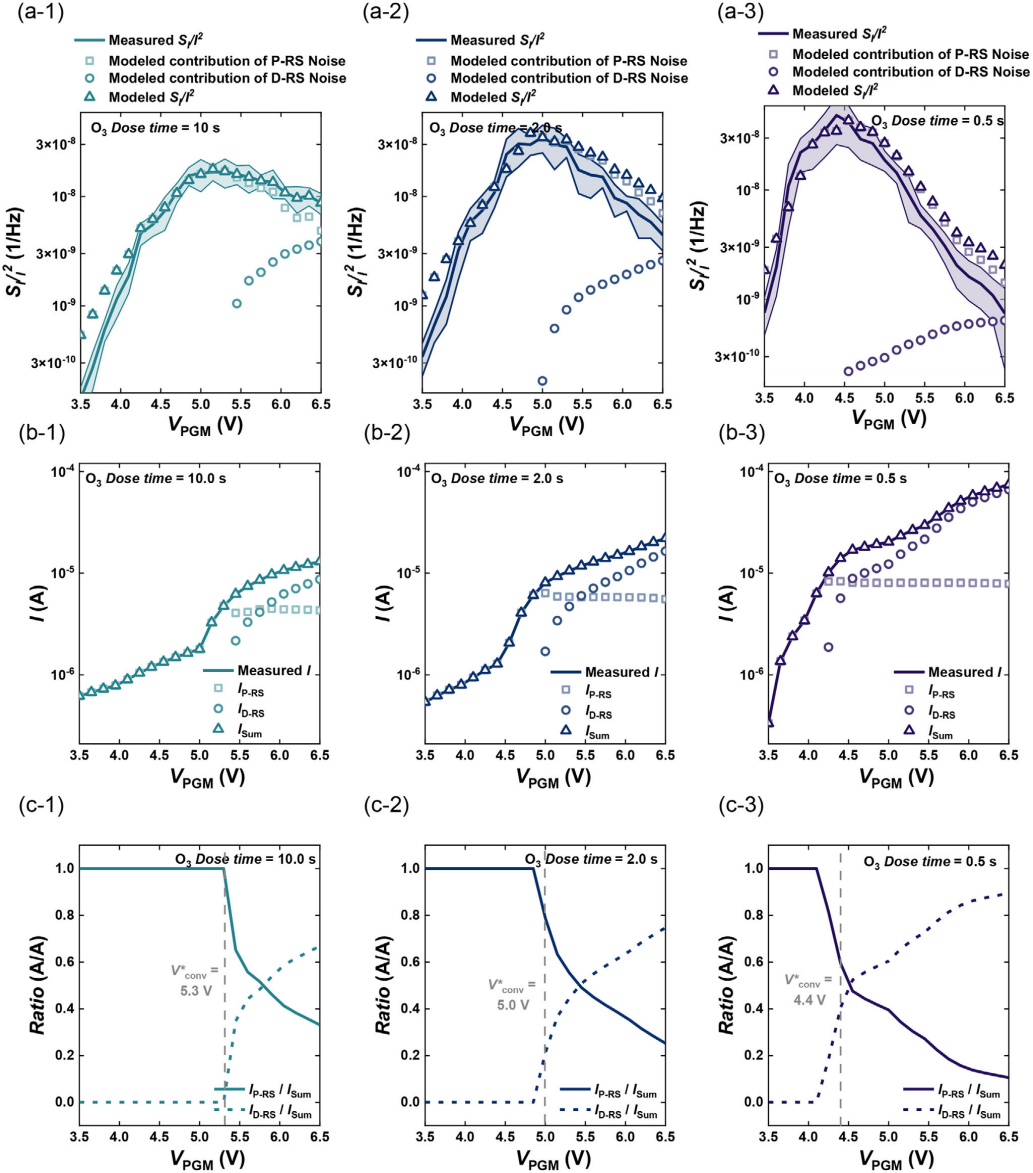
Noise‐fingerprint deconvolution results for devices fabricated with different O_3_ dose times. (a) Measured normalized noise (*S*_I_/*I* ^2^, line) versus *V*_PGM_ and corresponding fitting results, decomposed into contributions from P‐RS noise and D‐RS noise, along with their sum. (a‐1: O_3_ dose time 10 s, a‐2: O_3_ dose time 2 s, a‐3: O_3_ dose time 0.5 s). (b) Current decomposition results: measured current (line) versus *V*_PGM_ and fitting results showing P‐RS current, D‐RS current, and their total sum (*I*_SUM_). (b‐1: O_3_ dose time 10 s, b‐2: O_3_ dose time 2 s, b‐3: O_3_ dose time 0.5 s). (c) Extracted current fractions of P‐RS and D‐RS as a function of *V*_PGM_, demonstrating systematic redistribution of conduction from polarization‐mediated RS to defect‐mediated RS with increasing bias and reduced O_3_ dose. (c‐1: O_3_ dose time 10 s, c‐2: O_3_ dose time 2 s, c‐3: O_3_ dose time 0.5 s).

Figure 3a shows the schematic of the LFN measurement setup. A voltage bias is applied to the TiN/HfZrO/SiO_2_/*n* ^+^ Si stack, and the resulting current is monitored over time. The current is converted into a voltage signal by a low‐noise amplifier, which preserves the fluctuation spectrum, and the amplified signal is transformed into the power spectral density (PSD) by Fourier analysis. Together, the amplifier ensures sensitivity to small fluctuations, and the Fourier transform extracts the spectral signatures. Figure 3b presents the LFN spectra of devices with different O_3_ exposures: O‐rich HfZrO (10 s), O‐moderate (2 s), and O‐deficient (0.5 s). The noise data in Figures 3, 4, 5 were measured at a fixed read bias of *V* = 1.8 V. The corresponding *I* values for each noise measurement are provided in the inset. For each device, the normalized power spectral density (*S*_I_/*I* ^2^) is plotted as a function of *V*_PGM_. Across all *V*_PGM_ conditions, the spectra exhibit a characteristic 1/*f* dependence, while the overall magnitude of the noise varies systematically with *V*_PGM_. The extracted noise exponents γ(*V*_PGM_) are compiled in Figure S4, confirming that the spectra fall within the expected 1/*f* ^1^ regimes across processing conditions. In the O‐rich sample (Figure 3b), *S*_I_/*I* ^2^ increases with *V*_PGM_ at relatively low bias (3.5–5.3 V, Figure 3b‐1), characteristic of polarization‐mediated switching. This regime is consistent with Poole–Frenkel (PF) emission, where trapping–detrapping processes generate fluctuations that scale inversely with the electric field (∝1/*E*) [46], and enhanced fields during polarization reversal give rise to larger noise, consistent with prior ferroelectric noise reports [29]. Figure S5a illustrates the PF‐emission noise mechanism. Figure S5b shows ln(*J*/*E*) plotted versus *E* ^1/2^ in the LRS at different temperatures (293, 313, 333, and 353 K), where the data exhibit linear fitting behavior consistent with PF emission. Figure S5c shows ln[*J*/(*E*×*T* ^3/2^)] plotted versus 1/*T* at different electric fields (0.6, 0.7, 0.8, 0.9, and 1.0 MV/cm), which also yields linear fits, further supporting PF transport in this regime. These results support the use of the PF‐related interpretation for the operating regime discussed in Figure S5a. At higher voltages (5.35–6.75 V, Figure 3b‐2), *S*_I_/*I* ^2^ decreases with *V*_PGM_, signaling the onset of defect‐mediated switching. In this regime, migrating oxygen vacancies lower the effective barrier height and drive the device into a low‐resistance state. Similar suppression of normalized noise has been reported in HfO_2_‐based RRAM under vacancy‐driven conduction [29, 47]. The same rise–peak–fall trend is observed in the O‐moderate (2 s, Figure 3c) and O‐deficient (0.5 s, Figure 3d) samples: *S*_I_/*I* ^2^ increases due to P‐RS at low bias and decreases due to D‐RS at higher bias. This behavior is therefore a robust signature of the two switching mechanisms, regardless of O_3_ dose. What changes systematically with O_3_ dose is the transition bias (*V* ^*^), defined as the *V*_PGM_ at which *S*_I_/*I* ^2^ reaches its maximum. As O_3_ exposure decreases, *V* ^*^ shifts to lower bias and the post‐peak suppression becomes steeper, reflecting the stronger influence of vacancy‐mediated conduction in oxygen‐deficient films. Therefore, in addition to *V* ^*^, the magnitude of the slope of the log‐normalized noise provides a measure of how rapidly the transition proceeds, with steeper slopes indicating a larger contribution from defect‐mediated switching. To quantitatively characterize this crossover and compare different devices, we extracted two complementary metrics from the LFN spectra: the transition bias V* and the slope of the log‐normalized noise. The evolution of these metrics with O_3_ dose is summarized in Figure 4.

Figure 4a shows *S*_I_/*I* ^2^ versus *V*_PGM_ at 100 Hz for devices with O_3_ doses of 10, 2, and 0.5 s. Consistent rise–peak–fall topologies are also observed at 10 Hz and 1 kHz (see Figure S6), indicating that the crossover is insensitive to the analysis frequency. The curves are averaged across 10 devices (symbols: mean, shaded bands: ±1σ), and each exhibits a maximum that defines *V* ^*^. As the O_3_ dose decreases, *V* ^*^ shifts monotonically to lower bias and the post‐peak roll‐off steepens, consistent with earlier activation of vacancy‐mediated conduction.

To formalize this crossover, Figure 4b introduces the log‐slope metric, defined as:

(1) $$M\left(V\right)=d\left[\log\left(S_{I}/I^{2}\right)\right]/dV_{\mathrm{PGM}}$$

*M*(*V*) is positive in the P‐RS regime, negative in the D‐RS regime, and its zero‐crossing coincides with *V* ^*^. The magnitude |*M*| beyond the crossover grows as the O_3_ dose decreases, indicating a sharper redistribution toward defect‐mediated conduction in vacancy‐rich films. Together, the peak metric *V* ^*^ marks the bias at which the dominance switches, while *M*(*V*) captures both the direction and sharpness of the transition. Nevertheless, *V* ^*^ and *M*(*V*) on their own cannot be regarded as an absolute indicator. This is because devices differ in absolute current levels and *P*_r_, and the normalized peak position does not uniquely encode the underlying current fractions. As a result, *V* ^*^ reflects the apparent trend of the crossover but cannot reveal the true quantitative share of P‐RS and D‐RS. This fundamental limitation explains why earlier studies could identify the presence of P‐RS at low bias and D‐RS at high bias, yet remained blind to what happens in the intermediate regime. In other words, *V* ^*^ and *M*(*V*) are a convenient but insufficient metric—highlighting the need for a deconvolution framework that can explicitly extract the mechanism fractions across bias.

### 2.4 Noise‐Enabled Order‐Parameter Mapping of the P‐RS–D‐RS Transition

In order to move beyond qualitative identification of P‐RS at low bias and D‐RS at high bias, we developed a quantitative deconvolution framework that converts the measured noise into explicit polarization‐ and defect‐mediated contributions. We model the measured current (*I*(*V*_PGM_)) as the sum of two concurrent channels, polarization‐mediated current (*I*_P_(*V*_PGM_)) and defect‐mediated current (*I*_D_(*V*_PGM_)). Thus,

(2) $$I\left(V_{\mathrm{PGM}}\right)=I_{P}\left(V_{\mathrm{PGM}}\right)+I_{D}\left(V_{\mathrm{PGM}}\right)$$

According to the superposition rule for PSDs, the normalized noise can be expressed as a two‐source mixture. Defining the polarization current fraction:

(3) $$x\left(V_{\mathrm{PGM}}\right)=I_{P}\left(V_{\mathrm{PGM}}\right)/I\left(V_{\mathrm{PGM}}\right)\left(\mathrm{so}\;1-x=I_{D}\left(V_{\mathrm{PGM}}\right)/I\left.\left(V_{\mathrm{PGM}}\right)\right)\right.$$

And the mixture relation becomes [48]:

(4) $$\begin{array}{ccc} & & S_{n,\mathrm{tot}}\left(V_{\mathrm{PGM}}\right)\\ & & \;=S_{n,P}\left(V_{\mathrm{PGM}}\right)x{\left(V_{\mathrm{PGM}}\right)}^{2}+S_{n,D}\left(1-x\right.{\left.\left(V_{\mathrm{PGM}}\right)\right)}^{2}+2\rho x\\ & & \;\times \left(V_{\mathrm{PGM}}\right)\left(1-x\right.\left.\left(V_{\mathrm{PGM}}\right)\right){\left\{S_{n,p}\left(V_{\mathrm{PGM}}\right)S_{n,D}\left(V_{\mathrm{PGM}}\right)\right\}}^{1/2} \end{array}$$

where *S*_n,tot_(*V*_PGM_) is the measured normalized noise *S*_I_/*I* ^2^, *S*_n,P_(*V*_PGM_) and *S*_n,D_(*V*_PGM_) denote the normalized noise spectra of the polarization‐ and defect‐mediated channels, respectively, *x*(*V*_PGM_) is the polarization fraction, and *ρ* is the correlation coefficient between the two noise sources. In the main analysis, we set *ρ* = 0, i.e., we assume the P‐RS‐associated and D‐RS‐associated noise sources are uncorrelated, as a first‐order approximation to avoid introducing an additional free parameter that cannot be uniquely identified from *S*_I_ alone.

To obtain a physically consistent fit, the baseline construction and constraints were incorporated directly into the regression procedure. First, an initial estimate of *V* ^*^ was identified as the bias at which the measured *S*_n,tot_(*V*_PGM_) reached its maximum, consistent with the zero‐crossing of *M*(*V*_PGM_) introduced in Figure 4. Second, the lowest‐ and highest‐bias regions of the measurement window—where polarization‐mediated and defect‐mediated contributions respectively dominate—were taken as anchor points to seed the baselines *S*_n,P_(*V*_PGM_) and *S*_n,D_(*V*_PGM_). These extreme regions serve as reliable anchors that are minimally affected by the overlap of the two mechanisms. After anchoring, the intermediate portions were interpolated onto the full *V*_PGM_ range and guided by monotonic trends expected from the underlying mechanisms. Specifically, *S*_n,P_(*V*_PGM_) was constrained to increase or remain constant with bias below *V* ^*^, reflecting the growth of polarization‐mediated fluctuations, while *S*_n,D_(*V*_PGM_) was constrained to decrease or remain constant above *V* ^*^, consistent with the stabilizing effect of vacancy‐driven conduction. In this way, the anchored regions are extended across the entire bias range in a manner consistent with the physical behavior of each channel.

With *S*_n,P_(*V*_PGM_) and *S*_n,D_(*V*_PGM_) in place, the mixture law at each bias reduces to a quadratic equation in the polarization fraction *x*(*V*_PGM_) = *I*_P_(*V*_PGM_)/*I*(*V*_PGM_). The relation becomes:

(5) $$\begin{array}{ccc} x0(V_{\mathrm{PGM}}) & = & [\mathrm{Sn},D\left(V_{\mathrm{PGM}}\right)\pm \{\mathrm{Sn},D\left(V_{\mathrm{PGM}}\right)2-(\mathrm{Sn},P\left(V_{\mathrm{PGM}}\right)\\ & & +\;\mathrm{Sn},D\left(V_{\mathrm{PGM}}\right))\left(\mathrm{Sn},D\left(V_{\mathrm{PGM}}\right)-\mathrm{Sn},\mathrm{tot}\left(V_{\mathrm{PGM}}\right)\right)\}1/2]/\\ & & \times \;(\left(\mathrm{Sn},P\left(V_{\mathrm{PGM}}\right)+\mathrm{Sn},D\left(V_{\mathrm{PGM}}\right)\right) \end{array}$$

**SCHEME 1:**
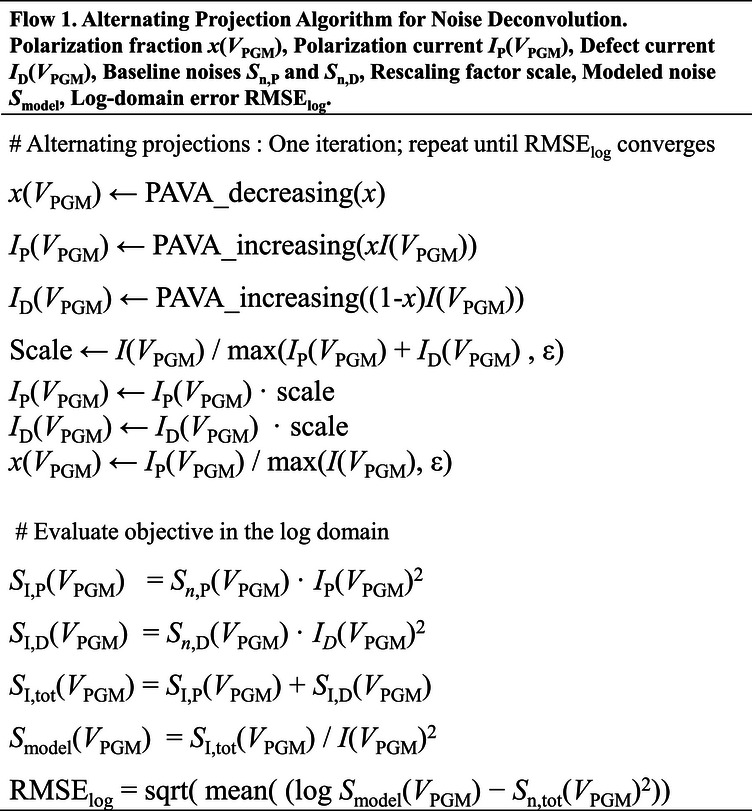
Alternating Projection Algorithm for Noise Deconvolution.

This quadratic provides the pointwise initialization of *x*(*V*_PGM_). Only solutions within the physical range [0,1] are considered admissible. The initial *x*_0_(*V*_PGM_) is regularized through an alternating projection procedure to enforce global physical consistency. Specifically, *x*(*V*_PGM_) is constrained to be non‐increasing with *V*_PGM_, while the partial currents *I*_P_(*V*_PGM_) and *I*_D_(*V*_PGM_) are constrained to be monotone increasing. Current conservation is then restored by minimal rescaling to satisfy *I*_P_(*V*_PGM_) + *I*_D_(*V*_PGM_) = *I*(*V*_PGM_). These monotonic constraints are implemented by isotonic regression using the Pool Adjacent Violators Algorithm (PAVA), which iteratively merges adjacent points that violate monotonicity and replaces them with their average, yielding the closest monotone sequence to the measured data [49, 50]. One iteration follows the workflow summarized in Scheme 1: *x*, *I*_P_, and *I*_D_ are sequentially projected onto their respective constraint sets and then rescaled to preserve current conservation.

After each cycle, the absolute noise components (*S*_I,P_ = *S*_n,P_· *I*_P_^2^ and *S*_I,D_ = *S*_n,D_·*I*_D_^2^) are reconstructed, the modeled normalized noise (*S*_model_ = (*S*_I,P_ + *S*_I,D_)/*I* ^2^) is updated, and the log‐domain error (RMSE_log_) is evaluated. Because each update is accepted only when RMSElog does not increase and the iteration stops at saturation, the final baselines and fractions are those that best reproduce the measured *S*_I_/*I* ^2^ under the dominance‐anchored baseline construction and physical constraints (representative convergence traces are shown in Figures S10 and S11). This process is repeated until RMSE_log_ converges, yielding smooth and self‐consistent profiles of *x*(*V*_PGM_), *I*_P_(*V*_PGM_), and *I*_D_(*V*_PGM_) that satisfy all physical constraints. With the method established, Figure 5a shows, for each O_3_ condition, the measured *S*_I_/*I* ^2^ (line) and the fitted decomposition into P‐RS noise and D‐RS noise, together with their sum (Figure 5a‐1: O_3_ 10 s; Figure 5a‐2: 2 s; Figure 5a‐3: 0.5 s). The convergence behavior of the alternating‐projection fit is summarized in Figure S7, where the RMSE_log_ trajectory versus epoch shows a monotonic decrease; the iteration terminates once further improvement falls below 1%. Representative convergence traces are provided in Figure S8 (RMSE_log_ versus epoch) and Figure S9 (RMSE_log_(*i*) / RMSElog(*i*+1) versus epoch) for O_3_ dose times of 0.5 s, 2 s, and 10 s, confirming monotonic error reduction and saturation at the stopping threshold. All traces in Figure 5a exhibit the characteristic rise–peak–fall topology, enabling quantitative extraction of the relative P‐RS and D‐RS noise contributions. The turning behavior can be interpreted by differentiating the uncorrelated mixture:

(6) $$\begin{array}{ccc} dS_{n,\mathrm{tot}}/dV_{\mathrm{PGM}\;} & = & \left(dS_{n,P}/dV_{\mathrm{PGM}}\right)\cdot x^{2}+\left(dS_{n,D}/dV_{\mathrm{PGM}}\right)\cdot {\left(1-x\right)}^{2}\\ & & +\;\left\{2xS_{n,p}\right\}\left(dx/dV_{\mathrm{PGM}}\right) \end{array}$$

where derivatives are with respect to *V*_PGM_ and *x*(*V*_PGM_) = *I*_P_(*V*_PGM_)/*I*(*V*_PGM_). Based on this relation, the contributions can be parsed term by term. First term (*dS*_n,P_/*dV*_PGM_)·*x* ^2^: in the polarization‐dominated region *dS*_n,P_/*dV*_PGM_ > 0, and the *x* ^2^ weight amplifies this positive contribution when the polarization current fraction is large. This term, therefore, drives *S*_n,tot_ upward as bias increases while P‐RS remains dominant.

In contrast, second term (*dS*_n,D_/ *dV*_PGM_)·(1−*x*)^2^: in the defect‐dominated region *dS*_n,D_/ *dV*_PGM_ < 0, and the (1−*x*)^2^ weight makes this negative contribution grow as the defect fraction increases. This term pulls *S*_n,tot_ downward at a higher bias. In addition, the third (coupling) term {2*xS*_n,P_ − 2(1−*x*)*S*_n,D_}(*dx*/*dV*_PGM_) reflects the redistribution of current fractions. Since *dx*/*dV*_PGM_ < 0 (the polarization share decreases with bias), and typically 2*xS*_n,P_ > (1−*x*)*S*_n,D_ around the transition (the polarization baseline dominates over the defect baseline), the product is negative. This adds an extra downward drive to *S*_n,tot_ as the system reweights from P‐RS to D‐RS. Crucially, the coupling term implies that the zero of *dS*_n,tot_/*dV*_PGM_ is not fixed by *x* alone. The peak V* balances three contributions—the baseline slopes *dS*_n,P_/*dV*_PGM_, *dS*_n,D_/*dV*_PGM,_ and the coupling term—so there is no requirement that *x*(*V* ^*^) = 0.5. For example, if the redistribution is slow (|*dx*/*dV*_PGM_| ≈ 0), the peak condition reduces to *x* ^2^ |*dS*_n,P_/*dV*_PGM_| ≈ (1−*x*)^2^ |*dS*_n,D_/*dV*_PGM_|, yielding *x* ≠ 0.5 whenever the slope magnitudes differ. Conversely, even if the slope magnitudes were comparable, a finite negative *dx*/*dV*_PGM_ and *S*_n,P_ > *S*_n,D_ shift the zero to *x* < 0.5. In short, the peak marks where the slope of the total noise changes sign, not where the two channels are equal. Thus, to determine the actual balance between P‐RS and D‐RS, one must examine the current fractions explicitly.

Conversely, with *dx*/*dV*_PGM_ < 0 and typically *xS*_n,P_ > (1−*x*)*S*_n,D_ near the transition, the coupling term is negative and shifts the zero to *x*<0.5. In short, the peak marks where the total‐noise slope changes sign, not where the two channels are equal; thus, the actual balance must be read from the recovered current fractions.

The decomposition shown in Figure 5a enables this, and Figure 5b then shows the current‐domain counterpart of the noise fit. The measured *I*(*V*_PGM_) (line) is reconstructed by the two partial currents *I*_P_(*V*_PGM_) and *I*_D_(*V*_PGM_), whose sum closely overlays the experimental data (Figure 5b‐1–b‐3). At low *V*_PGM_, *I*_P_ dominates; as *V*_PGM_ increases, *I*_D_ rises sharply and eventually overtakes *I*_P_. The bias at which this takeover begins follows the trend of *V* ^*^ extracted from the noise analysis, shifting to lower *V*_PGM_ as the O_3_ dose decreases. However, the numerical value of this current‐domain crossover (*I*_P_ = *I*_D_) does not coincide exactly with the V* defined only from the slope of the noise, highlighting the necessity of our deconvolution framework to determine the true mechanism fractions.

Finally, Figure 5c presents the deconvolution into mode fractions by plotting *x*(*V*_PGM_) (solid; P‐RS fraction) and 1− *x*(*V*_PGM_) (dashed; D‐RS fraction) for all processes (Figure 5c‐1–c‐3). These curves quantify the fraction of the total current carried by each mechanism at each bias. Because the analysis returns *x*(*V*_PGM_) and 1− *x*(*V*_PGM_) explicitly, we can report not only the shares *I*_P_(*V*_PGM_)/*I*(*V*_PGM_) and *I*_D_(*V*_PGM_)/*I*(*V*_PGM_) but also the absolute components (*I*_P_(*V*_PGM_), *I*_D_(*V*_PGM_)) and their noise counterparts (*S*_IP_(*V*_PGM_), *S*_ID_(*V*_PGM_)). To assess the sensitivity to the *ρ* assumption in Equation (4), we repeated the deconvolution using different *ρ* values. Since *ρ* is defined as Covariance(δ*I*_P_, δ*I*_D_)/(*σ*_P_ *σ*_D_), where *σ*_P_ and *σ*_D_ are the standard deviations of each fluctuation, it follows from the Cauchy–Schwarz inequality that Covariance(δ*I*_P_, δ*I*_D_) ≤ *σ*_P_ *σ*_D_. Therefore, by definition, *ρ* is strictly bounded as −1 ≤ *ρ* ≤ +1, and an actual device's *ρ* cannot exceed the range [−1, +1]. Figure S10 compares the extracted *x*_FE_ fraction as a function of *V*_PGM_ under different rho assumptions (for O_3_ dose times of 0.5, 2, and 10 s), confirming that the overall dominance‐shift trend is preserved. Figure S11 summarizes *V* ^*^ versus *ρ*, showing that *V* ^*^ decreases with increasing *ρ*, while the relative ordering across O_3_ dose conditions remains unchanged. In this way, Figure 5 demonstrates how the noise spectrum, previously treated only as a qualitative fingerprint, is elevated to a quantitative order parameter: a single mixture equation, anchored by physically constrained baselines and a two‐current model, captures the rise‐peak‐fall of *S*_I_/*I* ^2^, yields the mode fractions, and reveals how oxygen stoichiometry (via O_3_ dose time) shifts and sharpens the polarization‐to‐defect transition. Table 1 summarizes representative reports that discuss P‐RS and D‐RS interpretations in HfO_2_‐based ferroelectric stacks. Here, “direct vacancy control” refers to an experimental knob that intentionally and primarily modulates the oxygen‐vacancy concentration with a predictable direction, such that the vacancy level can be treated as an independent control variable. The table highlights how different studies have tuned processing conditions and interpreted the dominant switching mechanism.

**TABLE 1:** Comparison of representative prior works on the controversial interpretation of polarization‐mediated (P‐RS) and defect‐mediated (D‐RS) resistive switching in fluorite HfO_2_‐based devices.

Ref	LFN Spectroscopy	Switching Mechanism	Quantitative Seperation (P‐RS vs D‐RS)	Direct Vacancy Control	Design Guidelines
		P‐RS			
[51]	X	(Ferroelectric	X	X	X
		phase transition)			
		P‐RS			
[52]	X	(Tetragonal	X	X	X
		intermediate)			
[53]	X	D‐RS	X	X	X
		(Defect‐driven)			
[54]	X	D‐RS	X	X	X
		(Defect‐driven)			
		Mixed			
[29]	O	(P‐RS vs D‐RS)	X	X (Bias)	X
		Mixed			
[30]	O	(P‐RS vs D‐RS)	X	X (Anneal T)	X
		Mixed			
[31]	O	(P‐RS vs D‐RS)	X	X (Cycling damage)	X
		Mixed			
[32]	O	(P‐RS vs D‐RS)	X	X (Electrode type)	X
					O
This work	O	Mixed	O (Fraction map,	O	(first Vo‐linked design guidelines)
		(P‐RS vs D‐RS)	*x*_P_, *x*_D_)	(O_3_ Dose)	

## 3 Conclusion

In this work, we established a noise‐fingerprint deconvolution framework to quantitatively separate polarization‐mediated (P‐RS) and defect‐mediated (D‐RS) switching in hafnium oxide‐based ferroelectric films. Although the experimental validation in this study is performed on an FTJ platform, the proposed framework targets the switching‐mechanism duality of the ferroelectric thin film itself (P‐RS versus defect/vacancy‐mediated D‐RS) and can therefore be extended to other ferroelectric memory platforms. In particular, because our FTJ uses an MFIS stack that matches the gate‐stack structure of FeFETs, the same analysis can be applied using FeFET gate‐current (and noise) measurements, and likewise using leakage‐current (and noise) measurements in FeRAM‐type capacitors. By systematically varying the O_3_ dose time during deposition, we controlled the oxygen vacancy population and observed how the crossover from P‐RS to D‐RS evolves with programming bias. Across all process conditions, the normalized low‐frequency noise exhibited a reproducible rise–peak–fall signature, with the peak voltage (*V* ^*^) serving as a robust fingerprint of the transition between polarization‐ and defect‐dominated regimes. A central contribution of this study is the development of a physically constrained deconvolution methodology that transforms these noise fingerprints into quantitative mode fractions. By combining monotonic baseline fitting, mixture‐law solving, and alternating projection steps that enforce both fraction monotonicity and current conservation, we obtained self‐consistent polarization and defect current components together with their associated noise powers. This analysis revealed that the equal‐share point, where polarization and defect contributions become identical, shifts systematically to lower voltages as O_3_ dose time decreases. This finding provides direct evidence that oxygen stoichiometry dictates both the onset and the sharpness of defect‐mediated switching. Most importantly, this framework enables, for the first time, the quantitative determination of the bias‐dependent fractions of P‐RS and D‐RS, a task that had previously remained inaccessible. By moving beyond qualitative identification to explicit partitioning of the two contributions, our results provide a clearer answer to the long‐standing debate on the coexistence of polarization‐ and defect‐mediated switching in hafnia ferroelectrics. Practically, the extracted crossover voltage *V* ^*^ and the sign change of the slope metric *M*(*V*) provide a simple guideline to identify P‐RS‐dominant versus D‐RS‐dominant bias regimes, while the region around *V* ^*^ should be regarded as a mixed regime. In addition, the crossover systematically shifts with the vacancy‐control process knob (O_3_ dose time), offering a practical route to tailor the operating window through processing and to screen devices using noise‐based metrics when needed. We anticipate that this approach will not only resolve fundamental controversies but also offer practical guidelines for designing next‐generation ferroelectric memories and logic devices.

## 4 Experimental Section

### 4.1 Device Fabrication

Ferroelectric tunnel junctions (FTJs) were fabricated on *n* ^+^‑Si/SiO_2_ substrates. The HfZrO layer was deposited by ALD with alternating Hf and Zr cycles in a 1:1 ratio; precursor dose times for Hf and Zr were fixed, and only the O_3_ dose time was varied to 10, 2, and 0.5 s to tune oxygen stoichiometry. A TiN top electrode was formed subsequently, and rapid thermal annealing (RTA) was used to induce ferroelectricity.

### 4.2 Structural & Compositional Analysis

For depth‑resolved XPS, the TiN top electrode was removed by a peroxide wet etch (H_2_O_2_:H_2_O = 3:7, 60°C) to expose the HfZrO surface; measurements were performed with an ion gun energy of 4 keV. Binding energy was calibrated to C 1s (284.8 eV), a Shirley background was subtracted (23–13 eV window), and intensities were normalized to the Hf 4f7/2 peak. Spectra were deconvolved into Gaussian components (two doublets); the sub‑oxide ratio was extracted from fitted peak areas to quantify the O_3_‑dose dependence.

### 4.3 Electrical Characterization

PUND and Current–voltage (*I*–*V*) measurements were performed using a Keysight B1500A semiconductor parameter analyzer equipped with a pulse generator unit (PGU) and a waveform generator/fast measurement unit (WGFMU). The PUND sequence (ramp = 10 µs, top = 1 µs, ±5 V) was used to obtain polarization–voltage (*P*–*V*) hysteresis loops. *I*–*V* characteristics were measured at programmed voltages (*V*_PGM_) to evaluate the tunneling electroresistance (TER).

### 4.4 Low‑Frequency Noise (LFN) Measurements

Low‐frequency noise spectra were obtained by applying a constant bias to the device and recording the output current over time. The signal was amplified using a Stanford Research SR570 low‐noise current preamplifier and subsequently analyzed with an Agilent 35670A dynamic signal analyzer to extract the power spectral density (PSD). The normalized spectra (*S*_I_/*I* ^2^) were evaluated in the 4–1600 Hz range, unless otherwise specified.

## Conflicts of Interest

The authors declare no conflicts of interest.

## Supporting information




**Supporting File**: advs74252‐sup‐0001‐SuppMat.docx.

## Data Availability

The data that support the findings of this study are available from the corresponding author upon reasonable request.
