# Supplementary material for: Noise Fingerprints as a Quantitative Order Parameter for Polarization‐ and Defect‐Mediated Switching in Hafnia Ferroelectrics
Source: Adv Sci (Weinh). 2026 Feb 8;13(21):e19391. doi: 10.1002/advs.202519391 (PMC13073307; doi:10.1002/advs.202519391)
Supplement: Supplementary file 1 — Supporting File: advs74252‐sup‐0001‐SuppMat.docx. [file ADVS-13-e19391-s001.docx]

**Supporting information**

**Noise Fingerprints as a Quantitative Order Parameter for Polarization- and Defect-Mediated Switching in Hafnia Ferroelectrics**

Ryun-Han Koo^1^, Jiseong Im^1^, Joon Hwang^1^, Sung‐Ho Park^1^, Jonghyun Ko^1^, Kangwook Choi^1^, Sangwoo Ryu^1,2^, Gyuweon Jung^1^, and Jong-Ho Lee^1^

^1^Department of Electrical and Computer Engineering and Inter-University Semiconductor Research Center (ISRC), Seoul National University, Seoul 08826, Korea

^2^SK Hynix Inc, Icheon 17336, Republic of Korea

*Correspondence: jhl@snu.ac.kr (J.-H. L.)

Keywords: Noise spectroscopy, Hafnia ferroelectrics, Polarization-mediated switching, Defect-mediated switching, Low-frequency noise, Noise fingerprints

Figure S1. (a) AFM topography of the HfZrO surface; RMS and average roughness are indicated (*R*_q_ ≈ 1.87 nm, *R*_a_ ≈ 1.46 nm). (b) Extracted grain‑size distribution (histogram) for the HfZrO layer.

Figure S2. (a) PUND pulse scheme (ramp: 10 µs, top: 1 µs). (b–d) Transient current responses during the PUND sequence for O_3_ dose times of (b) 10 s, (c) 2 s, and (d) 0.5 s.

Figure S3. Endurance characteristics: 2*P*_r_ versus number of cycles for O_3_ dose times of (a) 0.5 s, (b) 2 s, and (c) 10 s.

Figure S4. Noise exponent γ (from *S*_I_∝1/*f* ^γ^) versus *V*_PGM_ for devices with O_3_ dose times of 10 s, 2 s, and 0.5 s; shaded bands mark the 1/*f* ^0^ (white), 1/*f* ^1^ (pink), and 1/*f* ^2^ (brown) regimes.

Figure S5. (a) Band‑diagram schematic of noise generation mechanisms in the TiN/HfZrO/SiO_2_ stack. (b) ln(*J*/*E*) plotted versus *E*^1/2^ at different temperatures (293, 313, 333, and 353 K), showing linear behavior consistent with PF emission. (c) ln[*J*/(*E*×*T*^3/2^)] plotted versus 1/*T* at different electric fields (0.6, 0.7, 0.8, 0.9, and 1.0 MV/cm), also showing linear fitting, further supporting PF emission in this regime.

Figure S6. Normalized low‑frequency noise (*S*_I_/*I*^2^) versus *V*_PGM_ for high (10 s), medium (2 s), and low (0.5 s) O_3_ dose times extracted at (a) 10 Hz and (b) 1 kHz.

**Figure S7.** Fitting performance of the proposed deconvolution algorithm: RMSE_log_ versus epoch for O_3_ dose times of (a) 0.5 s, (b) 2 s, and (c) 10 s.

**Fig. S8.** Convergence trace of the deconvolution algorithm (RMSE_log_ versus epoch). (a) O_3_ dose time = 0.5 s. (b) O_3_ dose time = 2 s. (c) O_3_ dose time = 10 s.

**Fig. S9.** Saturation criterion used for stopping (RMSE_log_(i) / RMSE_log_(i+1) versus epoch). (a) O_3_ dose time = 0.5 s. (b) O_3_ dose time = 2 s. (c) O_3_ dose time = 10 s.

**Fig. S10.** Sensitivity of the extracted *x*_FE_ fraction to the correlation coefficient ρ. *x*_FE_ fraction (*x*_FE_ = *I*_FE_/*I*) is plotted as a function of *V*_PGM_ for different assumed ρ values in Eq. (4). (a) O_3_ dose time = 0.5 s. (b) O_3_ dose time = 2.0 s. (c) O_3_ dose time = 10.0 s. While the absolute values vary slightly with ρ, the overall bias-driven shift from a P-RS-dominant regime to a D-RS-dominant regime is preserved.

**Fig. S11.** Extracted *V** versus the correlation coefficient rho for different O_3_ dose times. *V** is obtained from the deconvolution results under each ρ assumption and plotted for O_3_ dose time = 0.5 s, 2.0 s, and 10.0 s. *V** decreases with increasing ρ, indicating that the inferred crossover bias is moderately sensitive to ρ, while the relative ordering across O_3_ dose conditions remains unchanged.

Table S1. Bias-window summary from noise deconvolution
